# Guidelines for the management of a brain death donor in the rhesus macaque: A translational transplant model

**DOI:** 10.1371/journal.pone.0182552

**Published:** 2017-09-19

**Authors:** Tiffany J. Zens, Juan S. Danobeitia, Peter J. Chlebeck, Laura J. Zitur, Scott Odorico, Kevin Brunner, Jennifer Coonen, Saverio Capuano, Anthony M. D’Alessandro, Kristina Matkowskyj, Weixiong Zhong, Jose Torrealba, Luis Fernandez

**Affiliations:** 1 University of Wisconsin Department of Surgery, Division of Transplantation, University of Wisconsin School of Medicine and Public Health, Madison, Wisconsin, United States of America; 2 Wisconsin Primate Research Center, University of Wisconsin, Madison, Wisconsin, United States of America; 3 University of Wisconsin Department of Pathology, University of Wisconsin School of Medicine and Public Health, Madison, Wisconsin, United States of America; 4 University of Texas Southwestern Medical Center Department of Pathology, Dallas, Texas, United States of America; Fraunhofer Research Institution of Marine Biotechnology, GERMANY

## Abstract

**Introduction:**

The development of a translatable brain death animal model has significant potential to advance not only transplant research, but also the understanding of the pathophysiologic changes that occur in brain death and severe traumatic brain injury. The aim of this paper is to describe a rhesus macaque model of brain death designed to simulate the average time and medical management described in the human literature.

**Methods:**

Following approval by the Institutional Animal Care and Use Committee, a brain death model was developed. Non-human primates were monitored and maintained for 20 hours after brain death induction. Vasoactive agents and fluid boluses were administered to maintain hemodynamic stability. Endocrine derangements, particularly diabetes insipidus, were aggressively managed.

**Results:**

A total of 9 rhesus macaque animals were included in the study. The expected hemodynamic instability of brain death in a rostral to caudal fashion was documented in terms of blood pressure and heart rate changes. During the maintenance phase of brain death, the animal’s temperature and hemodynamics were maintained with goals of mean arterial pressure greater than 60mmHg and heart rate within 20 beats per minute of baseline. Resuscitation protocols are described so that future investigators may reproduce this model.

**Conclusion:**

We have developed a reproducible large animal primate model of brain death which simulates clinical scenarios and treatment. Our model offers the opportunity for researchers to have translational model to test the efficacy of therapeutic strategies prior to human clinical trials.

## Introduction

Since 1988, there have been over 669,000 solid organ transplants in the United States and almost 16,500 transplants were performed over the past year [1]. Currently, approximately 120,000 patients are on an organ transplant waiting list and 22 people die in United States daily while waiting for a transplant[1]. The scientific transplant community is constantly working to develop novel strategies to improve the quality of the available organ pool, as well as, extend the half-life of transplanted grafts. In order to accomplish these goals, animal models of transplantation are used to test new transplant drugs and patient treatment strategies.

Ideally, a model which mimics the cascade of events occurring after brain death would facilitate progress in the field of transplantation. Unfortunately animal models previously published have several deficiencies. First, most models described, use small animals (rats or mice) or non-primate large animals (swine or canines). Although scientific discoveries from these animal models had been tremendously valuable to the transplant community, the results gained from a large animal non-human primate model are more directly translatable to human subjects. Rhesus macaques’ DNA have a >90% similarity to humans[2]. Furthermore, primates possess equivalent HLA-A, HLA-B, HLA-E, HLA-F and HLA-G genes which are critical to understanding the immunologic barriers in transplantation in regards to the human histocompatibility complex[3]. Given the biological similarities with regards to shared anatomy, response to physiologic stress, and drug metabolism between humans and non-human primates, this model provides a significant advantage over rodent or porcine models previously characterized in the literature [4–6].

Second, many of the current brain death animal models are not able to replicate a clinical scenario given the typical duration of time from declaration of brain death to organ procurement is too long to be reproduced in a small animal model. Brain death is associated with hemodynamic instability, hormonal changes, a systemic release of pro-inflammatory cytokines, complement activation and derangement in the coagulation system. It has been established that these physiologic changes result in damage to the transplantable organs that unfortunately translate into shorter graft survival [7–9]. With the exception of a single study by *Sereinigg et al*, most brain death experiments last between 4–10 hours[10–15]. In contrast, the average time from human brain death declaration to organ procurement is 20–30 hours [16, 17]. Therefore, a model that recapitulates a similar time interval is required to elucidate the factors which damage organs during brain death and the degree by which organ damage translates to poorer outcomes when compared to living donors.

The final limitation in traditional brain death animal models is that the standard of care provided by an Intensive Care Unit (ICU) staff to brain dead human donors has been impossible to reproduce in smaller species. It is known that quality ICU care can decrease the deleterious physiologic consequences of brain death on the transplantable organs, improve the percentage of organs accepted for transplant, and improve outcomes of those grafts [18–24]. Thus, a clinically translatable model needs to reproduce this setting, implementing modern treatments in response to the donor’s medical state.

In order to create the optimal brain death animal model to study transplant treatments and medications, one needs to address all of these issues. Over the last year, we have been working on a non-human primate 20-hour brain death transplant model which provides ICU level care to these animals and can be used to test exciting and innovative medical and technical advances in transplantation. In this paper, we will outline our methodology so it can be applied and utilized by other transplantation laboratories and scientists studying the effects of human brain death, the biology of organ donation and severe traumatic brain injury.

## Materials and methods

Rhesus macaques used in this study were housed at the Wisconsin National Primate Research Center, University of Wisconsin Madison, in accordance with Institutional Animal Care and Use Committee (IACUC) guidelines. The experiment was conducted under the University of Wisconsin School of Medicine and Public Health IACUC approved protocol. The animals are house either individually or paired in cages meeting the animal welfare guidelines for cage sizing. They are on a 12 hour light/dark cycle, temperature maintained at 64-84F and humidity at 30–70%. The animals for this experiment are part of the specific pathogen free colony and are negative for SRV, STLV, SIV, and Herpes B. They are fed approximately 6–9 biscuits of Teklad Global 20% Protein Primate Diet twice daily. Prior to surgical procedures, animals are food deprived for approximately 4–18 hours. Water is provided continuously via an automatic water with lixit system. The primate enrichment program include a wide variety of produce and foraging items provided once daily. All animals are monitored a minimum of twice daily. Analgesia is provided with narcotics for procedures and until brain death confirmation, they are under general anesthesia. After brain death, the animal is unable to experience pain, so anesthesia is removed for the maintenance period. Although the animal is still unable to detect pain, for the procurement laparotomy, they are placed back on isoflurane general anesthesia as a precautionary measure. For our experiment, while under general anesthesia, the animals are sacrificed by exsanguination. The animals were continuously monitored throughout the experiment. Chlorohexidine or betadine prep was used prior to all invasive and sterile procedures.

### Cardiac monitoring

Cardiac monitoring was displayed in real time to a portable Advisor (R) Vital Signs Monitor by SurgiVet (Smiths Medical, OH). ECG electrodes on the animals’ hands and feet displayed continuous heart rate and rhythm. Continuous hemodynamic monitoring was obtained with the use of femoral arterial line and femoral central venous pressure (CVP). A femoral cut down was performed to place the arterial and central line. The vein was accessed and a 5.5F, triple lumen, 30cm long pediatric ARROW central line was placed using the Seldinger technique. Next, an arterial line was placed using a 4F, 12 cm long Cook arterial line. Both lines were tested for brisk blood return. The arterial line and brown port of the central line were connected to transducers in order to display continuous arterial and CVP waveforms respectively.

### Respiratory monitoring

End tidal CO2 (ETCO2) capnography was attached to the endotracheal circuit and displayed continuously throughout the experiment. SpO2 monitoring was achieved by applying SpO2 monitors to the animal’s fingers, toes or buccal mucosa.

### Other monitoring

Hourly urine output was monitored by inserting an 8F Bard Foley catheter and attaching a urinometer. Continuous temperature monitoring was measured through an esophageal temperature probe. Vital signs were recorded on a spreadsheet every 15 minutes. Net volume status calculations (total volume in-total volume out) were calculated every hour to avoid dehydration or fluid overload.

### Laboratory values

All laboratory tests were monitored with an Istat (Abbot, Princeton, NJ) device. Chemistry labs were obtained using a Chem8 (sodium, potassium, chloride, ionized calcium, CO2, BUN, creatinine, glucose, hemoglobin and hematocrit) cartridge, Arterial blood gases (ABG) results obtained using a CG4 (pH, CO2, pO2, HCO3, base excess, lactate, and oxygen saturation) cartridge and troponin results obtained using a cTnI (troponin) cartridge. Arterial blood gases and chemistry lab panels were drawn every 2–4 hours as needed and troponin levels checked every 6 hours until they peaked.

### Neurologic

#### Induction of brain death

After baseline laboratory values were obtained, brain death was induced. Using a 2mm Dremel rotary tool, a Burr hole was made through the skull exposing the animal’s dura mater. The dura mater was then carefully dissected away from the skull without puncturing it and a 14F Foley catheter was then inserted between the skull and the dura mater. The catheter balloon was inflated with 7-9ml of saline solution and the animal was monitored for the expected hemodynamic instability of brain death in a rostral to caudal fashion. First, at the level of the medulla, there was a sympathetic surge in order to maintain cerebral perfusion pressure that results in profound tachycardia and hypertension. Next, spinal cord ischemia and herniation resulted in deactivation of the sympathetic nervous system, decreased catecholamines, and vasodilation causing normalization of heart rate and hypotension.[19] After this hemodynamic instability was documented, the isoflurane anesthetic is turned off and confirmation of brain death followed.

#### Confirmation of brain death

Confirmation of brain death was achieved according to the current recommendations by the American Academy of Neurology (AAN) in 1995[18]. For confirmation, the isoflurane must be off for at least 30 minutes and the animal may not have received any narcotics, paralytics or benzodiazepines. The animal body temperature must be normal prior to declaration of brain death and there can be no severe acid/base or electrolyte disturbances.

In order to confirm brain death, we first performed a physical exam. Brain dead animals manifested fixed and dilated pupils, absent corneal, gag, and cough reflexes, and no movement of the limbs to painful stimuli. Next, an apnea test was conducted. The ventilator was turned off for between 8–10 minutes. The animals were monitored for any spontaneous breaths. If no breaths were documented, an arterial blood gas was drawn and the ventilator turned back on. If a PCO2 was documented as 60mmHg or 20mmHg above the baseline, brain death was confirmed. If the animal’s SpO2 dropped below 85% during the apnea challenge with no spontaneous breaths, an ABG was drawn and the animal placed back on the ventilator. In concordance with the AAN, no ancillary testing (cerebral angiogram, electroencephalogram (EEG), transcranial Doppler) was needed in the presence of a positive apnea test and physical exam consistent with brain death.

### Cardiac

#### Hemodynamic maintenance: IV fluid therapy and vasoactive agents

Over the 20-hour maintenance period of brain death, the hemodynamic goals were to maintain a mean arterial pressure (MAP) greater than 60mmHg and heart rate (HR) within 20 beats per minute (bpm) of baseline HR. In order to accomplish, this we utilized a variety of cardiac medications in accordance with current human critical care guidelines (Table 1)[18–20, 25, 26].

**Table 1:** Cardiac medication management.

Drug	Mechanism/Indication	Infusion rate
Isotonic fluids	Increase preload	- 10ml/kg bolus LR or 0.9NS up to x 4 initially and then guided by volume losses (urine output)
Maintenance intravenous fluids (MIVF)	Replace insensible losses	- Use “4-2-1” rule - May use 0.9NS initially, change to 0.45NS if Na>145 - May stop MIVF is total fluids from drips exceeds maintenance rate
Labetalol	Non-selective beta adrenergic blocker, used to treat persistent tachycardia or hypertension and decrease myocardial oxygen demand	- 0.25–0.5 mg/kg IV push as needed every 1 hour
Atropine	Muscarinic acetylcholine receptor antagonist used for bradycardia	- 0.02 mg/kg IV up to max dose of 0.5mg
Potassium	Used for electrolyte replacement	- 1 mEq/kg IV every one hour as needed for K+ <3.2
Calcium Gluconate	Used for electrolyte replacement	- 50 mg/kg IV every 4 hours as needed for iCa<1
Vasopressin	A potent vasoconstrictor via the V1 receptor in vascular smooth muscle and enhances fluid reabsorption in the renal collecting system via V2 receptors. **First line drug** for **hypotension** when evidence of **diabetes insipidus** (DI).	- Continuous infusion: dose 0.0003–0.005un/kg/min
Dopamine	Dopaminergic effects at 0.5–2 mcg/kg/min, beta 1 adrenergic agonist effects at 2–10 mcg/kg/min, and alpha adrenergic effects at >10 mcg/kg/min. Used as **first line** infusion for **hypotension** if **no evidence of DI** second line if evidence of DI	- Continuous infusion 5-50mcg/kg/min. If dose exceeds 10mcg/kg/min, consider second agent.
Dobutamine	Direct inotropic agent that stimulates beta receptors in the heart. **Third line drug** for **hypotension** if animal has **evidence of significant myocardial infarction** or ventricular compromise	- Continuous infusion 0.5–20 mcg/kg/min
Epinephrine	Alpha-1, Alpha-2, Beta-1, Beta-2 adrenergic agonist. **Third line drug** for **hypotension** when **no evidence of significant myocardial infarction** or ventricular compromise	- Continuous infusion at 0.1–1 mcg/kg/min

**Cardiac medication management:** Medication indication, mechanism and dosage for IV medications used in brain death model.

Maintenance IV fluids (MIVF) were administered to each animal to replace insensible losses. MIVF were calculated using the “4-2-1” rule with 4ml/kg/hour for the first 10kg + 2ml/kg/hour for the second 10 kg + 1ml/kg/hour for the remainder. Initially, isotonic fluids of 0.9% normal saline (0.9NS) are used for MIVF. If sodium (Na) was greater than 145, 0.45% normal saline (0.45NS) was used given these animals are prone to hypernatremia. Intravenous (IV) fluid replacement was primarily driven by central venous pressure (CVP) and hourly net total volume calculations. Our goal was to have a net even volume status for the experiment with a CVP< 6mmHg.

**Fig 1:**
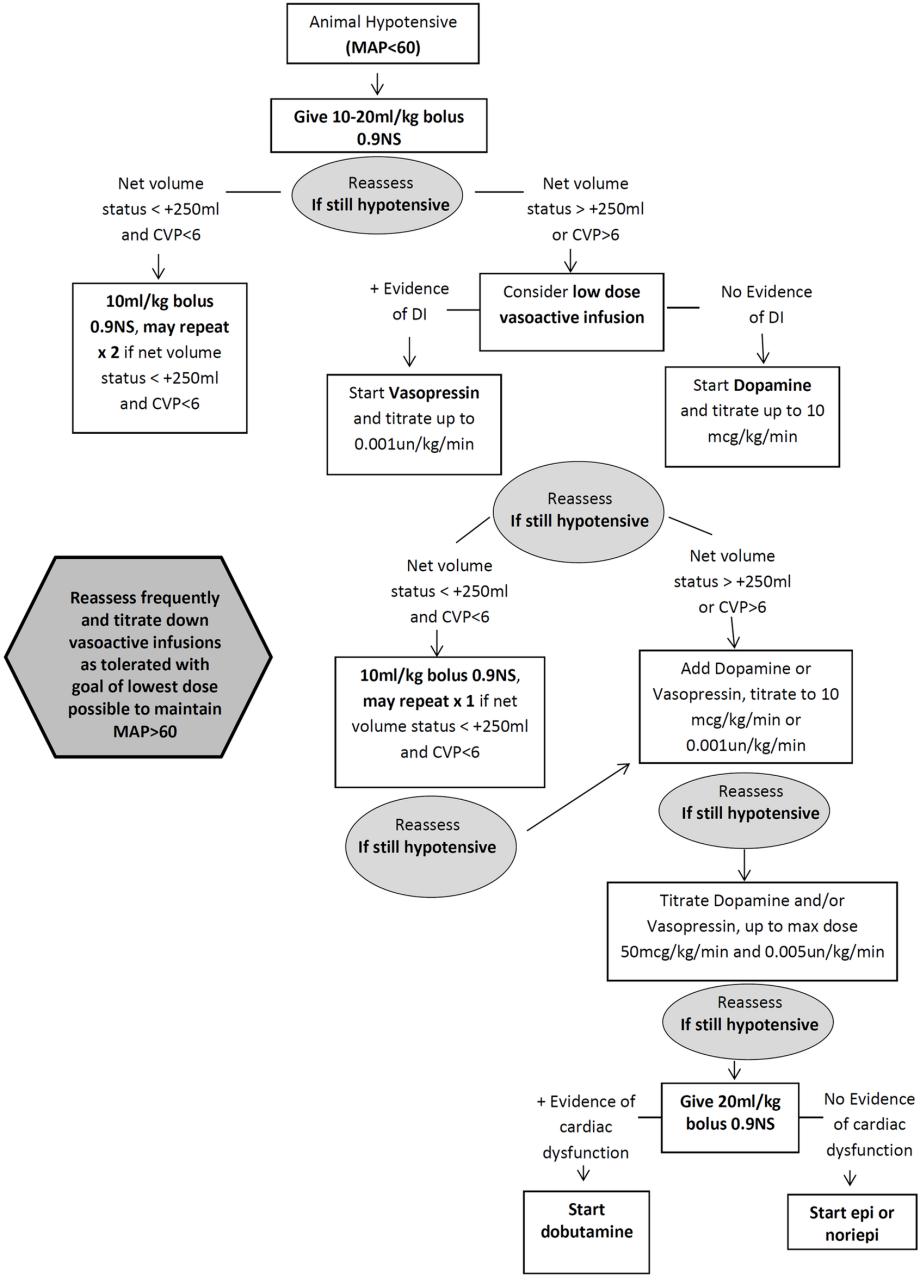
Algorithm for management of a hemodynamically unstable primate. An algorithm for vasoactive infusions and isotonic IV fluid boluses needed to augment the animal’s blood pressure is outlined above.

An algorithm for vasoactive infusions and isotonic IV fluid boluses needed to augment the animal’s blood pressure is outlined in Fig 1. The choice of first line vasoactive medication was dependent on whether the animal showed evidence of diabetes insipidus (DI). If the animal had polyuria or hypernatremia, vasopressin was used as a first line drug and titrated to max dose of 0.001units/kg/min before adding dopamine as a second agent. Dopamine can be used as the first line drug in animals without evidence of DI. In these cases, if the dopamine infusion exceeded 10mcg/kg/min, consider adding vasopressin as a second agent [25, 26]. Epinephrine infusion or norepinephrine infusion can be used as a third line option for profound hypotension if there is no evidence of cardiac compromise. In animals with evidence of significant myocardial infarction (MI) or ischemia on electrocardiogram (ECG) or elevated troponin values, dobutamine should be used as the third line drug and epinephrine/norepinephrine used as a fourth line last resort medication.

In instances in which the animal exhibited persistent (>1 hour) tachycardia (>20 bpm over baseline) and hypertension (SBP>145mmHg), low dose labetalol IV push was administered in order to minimize the deleterious cardiac consequences of the increased myocardial oxygen consumption[26]. In cases of persistent bradycardia (HR<70bpm) atropine was administered. In addition, electrolytes (particularly potassium and calcium) were monitored and replaced during brain death.

### Respiratory

#### Anesthesia induction and mechanical ventilator management

Animals were pre-medicated prior to intubation with 15mg/kg of ketamine, 0.4mg/kg of atropine and 0.01–0.02 mg/kg of buprenorphine. Using a laryngoscope with a Macintosh blade, the animals were intubated with the appropriate size endotracheal tube (usually 4.0 or 4.5). The tube was secured in place and verified via end tidal CO2 (ETCO2) and bilateral breath sounds.

Animals were mechanically ventilated using a pressure controlled ADS2000 ventilator (Engler, Hialeah, Florida). Standard starting ventilator settings were FiO2 = 100%, respiratory rate 8–10 breaths/min, flow rate 6L/min, peak inspiratory pressure = 10–12. Arterial blood gases (ABGs) were measured every 4 hours to determine if changes were needed in the ventilator settings (Table 2).

**Table 2:** Interpretation of ventilation and oxygenation disturbances on ABG.

Typical Acid- Base Disturbance	Intervention	Comments
Normal ABG: pH = 7.35–7.45, PCO2 = 35-45mmHg, PaO2>80mmHg, HCO3 = 22–26		
If animal is acidotic and PCO2>45	Increase ventilator respiratory rate or tidal volume	
If animal is alkalotic and PCO2<35	Decrease ventilator respiratory rate or tidal volume	
If animal is hypoxic with PaO2<80 and lung sounds course	In-line suction with 10F sterile suction catheter Increase FiO2 if not at 100%	If animal has a significant amount of thin secretions and little improvement with suctioning (SpO2<90% after 30 min), consider pulmonary edema as a possible etiology. Animal may improve with 2–4 mg/kg single dose Lasix
If animal is hypoxic with PaO2<80 and lung sounds course	Do 10 bag-hold breaths with pediatric Ambu-bag to promote recruitment of alveoli. You may also consider attaching PEEP valve to Ambu-bag and administering 5-10mmHg of PEEP. Increase FiO2 if not at 100%	Consider alveolar hypoventilation as cause for hypoxia

**Interpretation of ventilation and oxygenation disturbances on ABG:** Trouble-shooting techniques for maintaining adequate ventilation and oxygenation in the brain death model

### Endocrine

#### Diabetes insipidus (DI): Inadequate secretion of an anti-diuretic hormone as a result of brain death

Diabetes insipidus characterized by a urine output >4 ml/kg/hr with a high serum sodium >145 mEq/L, and increased serum osmolality >300 mosmol kg^−1^, and a low urine osmolality <200 mosmol kg^−1^. DI management included early use of vasopressin and fluid replacement with solutions containing minimal sodium.

Continuous vasopressin infusions at a dose of 0.0003un/kg/min-0.005un/kg/min provided a synthetic replacement for the lost endogenous hormone. In addition, isotonic fluid boluses guided by CVP and hourly net fluid volume calculations were used to prevent dehydration and contraction alkalosis. Even with appropriate use of vasopressin infusions and volume resuscitation, some animals developed hypernatremia from their DI. In cases in which sodium (Na) levels exceed 150mEq/L, 5% dextrose in water (D5W) infusions were necessary to prevent severe hypernatremia, which has been associated with worse transplant outcomes[19, 25]. The infusion rate for D5W infusions was calculated using the Adrogue formula for sodium correction.

#### Hyperglycemia

Pro-inflammatory conditions and physiologic stress is often associated with hyperglycemia. Hyperglycemia can cause osmotic polyuria and worse pre-recovery renal function[17, 25]. Hypoglycemia should also be avoided. As a result, a target glucose of <200mg/dl and >60mg/dl was implemented for this experiment.

### Organ procurement

After 20 hours, the animal’s abdomen was prepped and draped for organ procurement. A midline laparotomy was performed and the colon mobilized to expose the retroperitoneum. The left and right kidneys and the ureter were dissected free from the neighboring structures with the adrenals left in place. The infra-renal aorta was mobilized and 2.0 silk sutures looped circumferentially at the proximal and distal segments of the inferior mesenteric artery which often was ligated and divided to gain larger lengths. The inferior vena cava (IVC) was mobilized and dissected free to visualize both renal veins and the infra-hepatic inferior vena cava. The proximal supraceliac aorta was dissected an vessel loop placed around the artery in order to facilitate its clamping After making an arteriotomy in the infra-renal aorta, an angiocath of appropriate size (typically 14G-18g) was placed into the lumen of the abdominal aorta and secured in place. 10,000 units of heparin was administered intravenously. The supreceliac aorta was then clamped. Next, 1 liter of UW solution was infused into the abdominal organs via the aortic cannula and the distal IVC was incised, allowing the animal to exsanguinate. The abdomen was packed with sterile ice. Organs for transplantation and research were retrieved using conventional surgical techniques.

Following organ procurement, 20G core needle biopsies were taken from the kidneys and wedge biopsies taken from the liver and pancreas. H&E histological analysis was performed on these specimens by board certified pathologists to determine any damage sustained to the organ during brain death. Given these organs were from elderly animals, naïve elderly renal, pancreatic, and hepatic tissue was obtained for comparison to determine the baseline level of organ damage in a geriatric animal. Additionally, the Remuzzi score[27] was used to determine the transplant eligibility for each kidney biopsy. The Remuzzi score is used by pathologists and transplant surgeons to determine if expanded criteria kidneys should be considered for single organ transplant, dual kidney transplant or discarded. A score of 0–3 indicates an organ is amenable for single kidney transplant, a score of 4–6 indicates dual kidney transplant should be considered and a score of 7–12 indicates the organ should not be transplanted.

## Results

A total of nine rhesus macaque animals were used in this model. The mean age of the animals was 17.9±1.41years and the mean weight was 8.07±1.61kg.

### Neurologic

#### Confirmation of brain death

**Fig 2:**
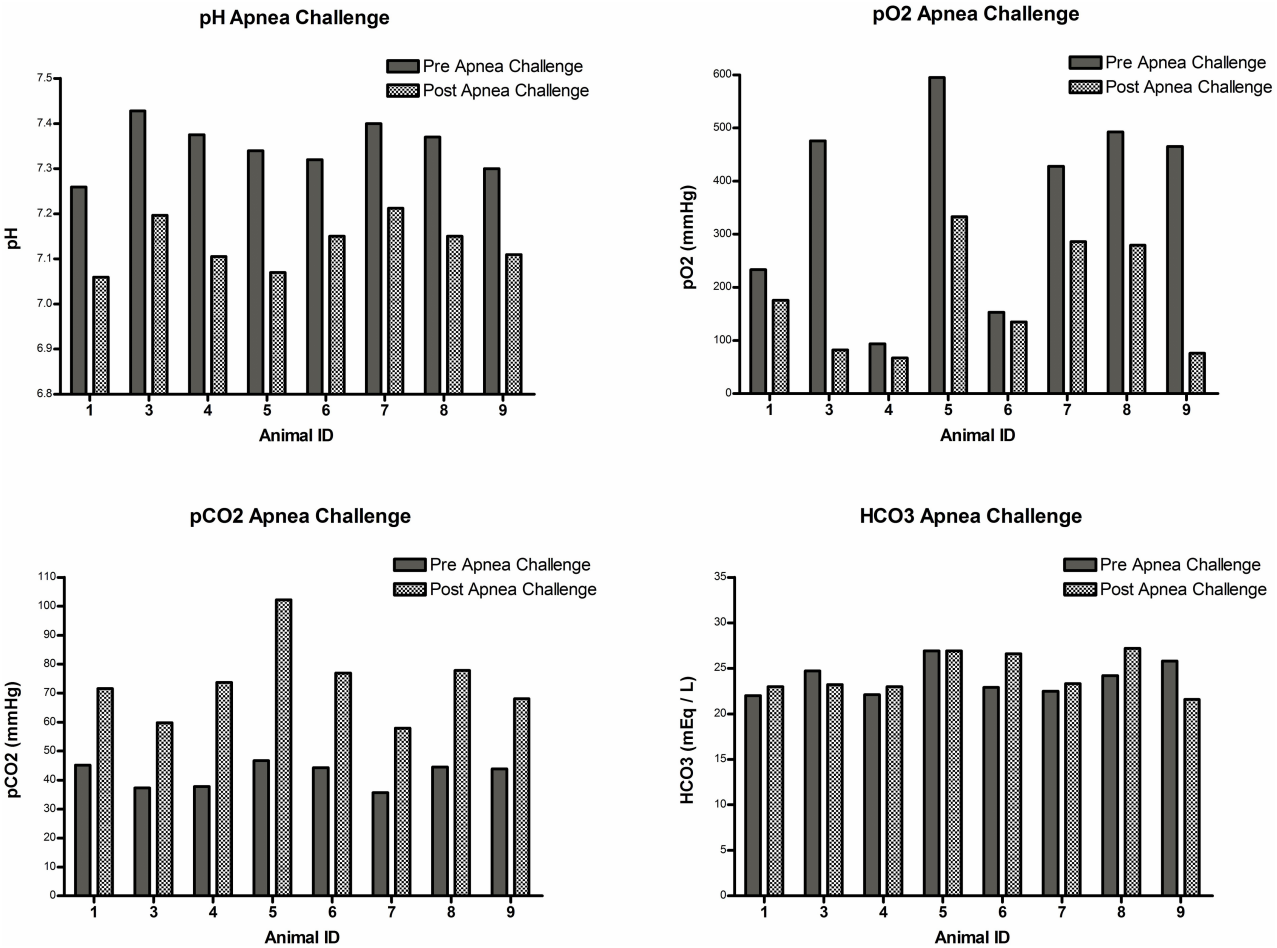
Confirmation of brain death: Apnea challenge. All but one animal (#2) were stable enough from a respiratory standpoint to undergo a successful apnea challenge. All animal experienced respiratory acidosis and a rise in pCO2 of greater than 20mmHg without a spontaneous breath, consistent with brain death. Other than the single animal who became extremely hypoxic, the remaining animals were able to maintain pO2>70 during the challenge with pre-oxygenation techniques.

Prior to performing the apnea challenge and clinical exam to confirm brain death, the animals had baseline labs and vital signs to ensure they were normothermic and without any significant acid/base or electrolyte abnormalities in compliance with the AAN guidelines (summarized in Table 3). Physical exams performed off anesthesia were consistent with brain death (absence of reflexes and no response to pain) in all animals. Eight of the nine animals were stable enough from a respiratory standpoint to undergo an apnea challenge. The remaining animal (#2) continued to desaturate to SPO2<80% when taken off the ventilator. The apnea challenge results are summarized in Fig 2. All animals experienced respiratory acidosis and a rise in pCO2 >20mmHg without a spontaneous breath, consistent with brain death. Other than a single animal (#2) who became extremely hypoxic, the remaining animals were able to maintain pO2>70 during the challenge with pre-oxygenation techniques.

**Table 3:** AAN guidelines prior to confirmation of brain death.

AAN Criteria Prior to Brain Death Declaration	Brain Death Model Animals (n = 9)
Normothermia or mild hypothermia (temp>36°C)	Maximum temperature: 38.0°C Minimum temperature: 36.2 C Mean temperature: 37.0± 0.57°C
Absence of severe acid-base abnormality	Maximum pH: 7.43 Minimum pH: 7.26 Mean pH: 7.36±0.05
Absence of severe electrolyte abnormality	Maximum potassium: 3.6 mEq/L Minimum potassium: 2.9 mEq/L Mean potassium: 3.2±0.2 mEq/L Maximum sodium: 155 mEq/L Minimum sodium: 145 mEq/L Mean sodium: 147.33±3.08 mEq/L Maximum chloride: 119 mEq/L Minimum chloride: 109 mEq/L Mean chloride: 112.56±3.36 mEq/L

Baseline laboratory tests and vital signs confirmed compliance with AAN guidelines prior to proceeding with testing to confirm brain death

### Cardiac

#### Initial hemodynamic instability

**Fig 3:**
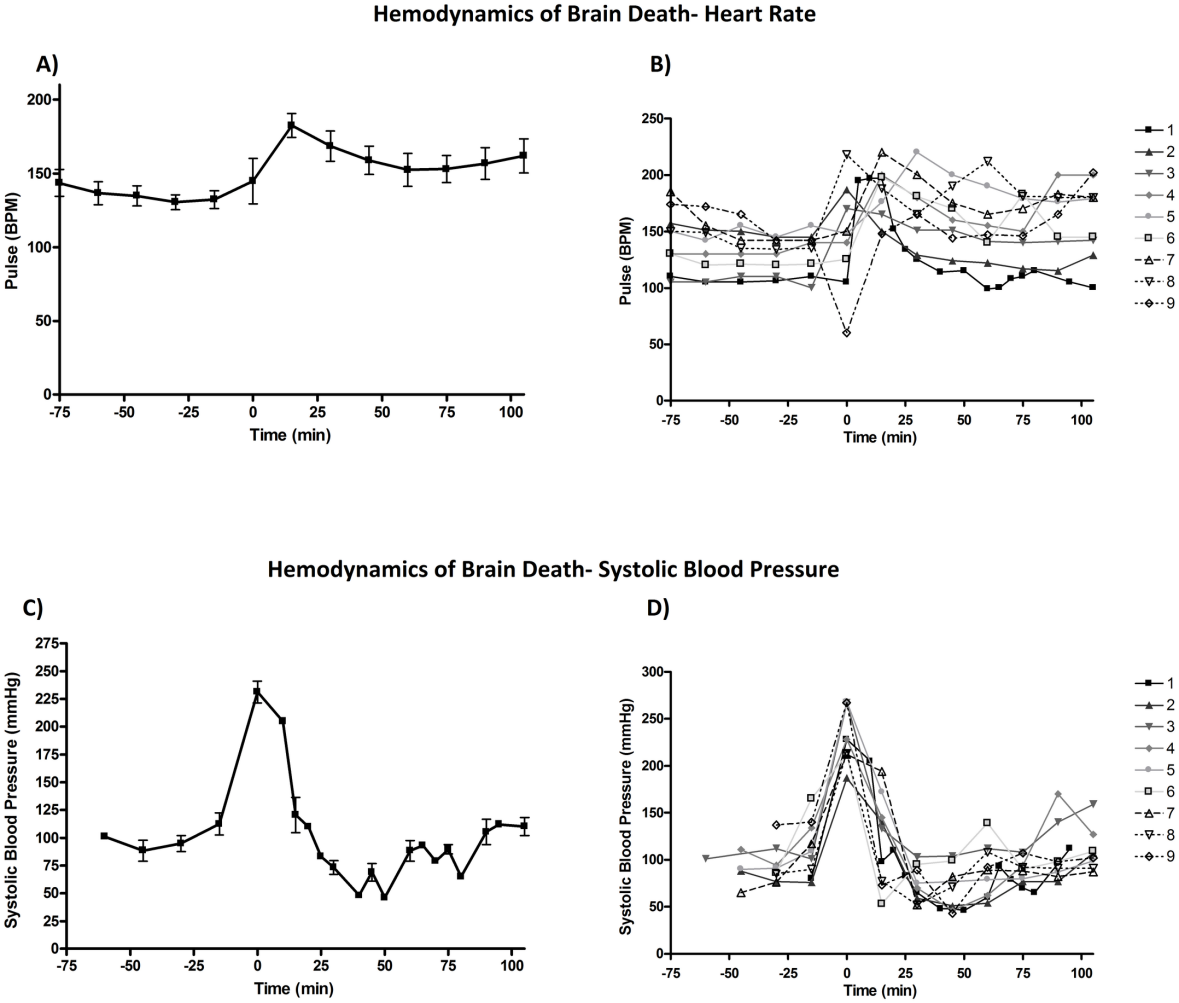
Hemodynamics of brain death: Systolic blood pressure and heart rate. When examining the change is systolic blood pressure (SBP), there was a predicable hypertension, followed by hypotension before return to baseline. Fig 3B shows the individual SBP response to brain death and Fig 3A shows the average response for all animals. When change in HRs with BD there was a trend of slightly delayed tachycardia at 15 minutes after brain death, followed by slow return to a HR approximately 10–15 bpm above the animal’s original baseline by 1 hour post brain death. Fig 3C shows the individual HR response to brain death and Fig 3D shows the average response for all animals.

**Fig 4:**
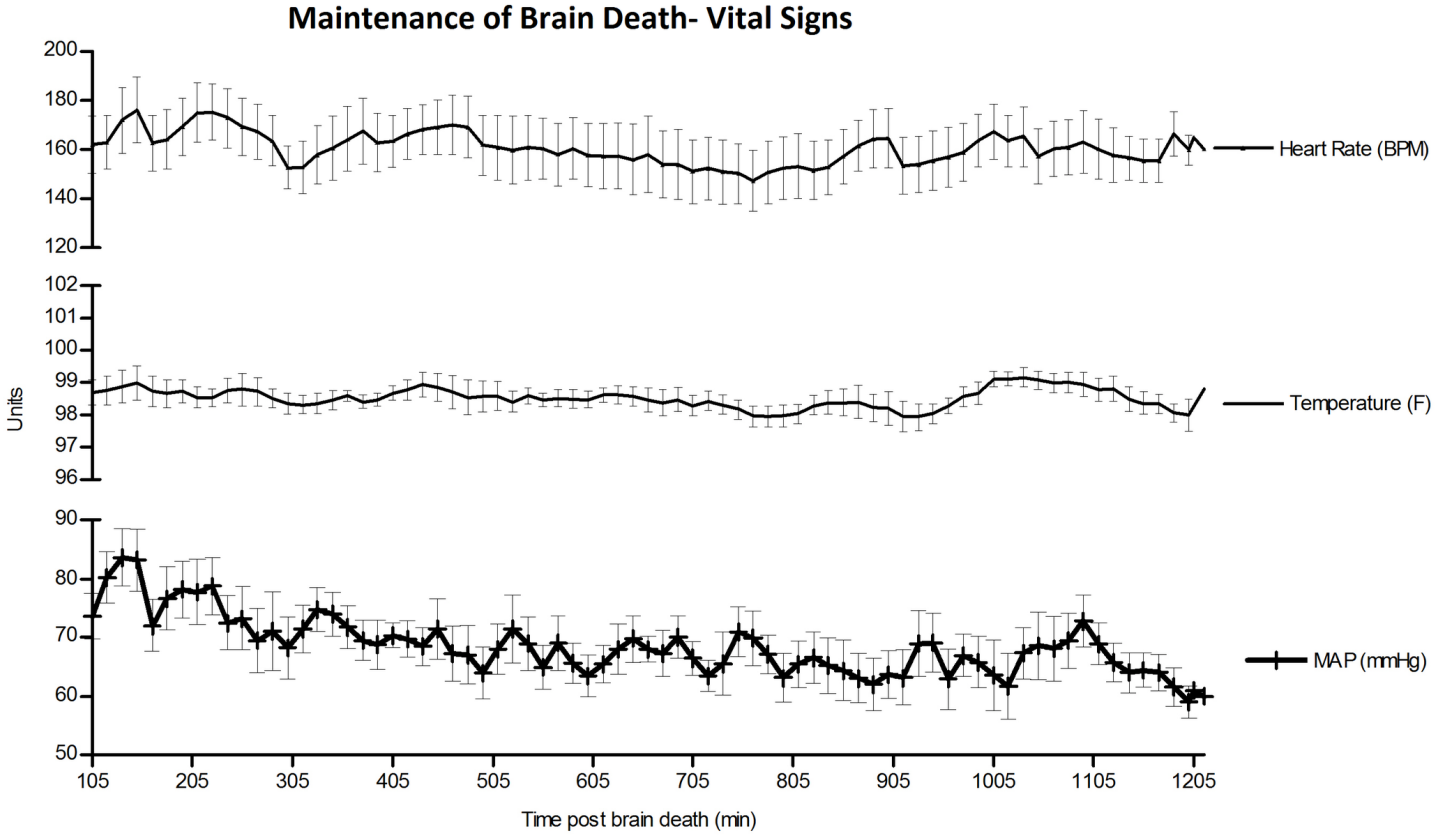
Maintenance of brain death—Vital signs. We were able to maintain hemodynamic stability throughout brain death with MAP>60 and HR within 20 bpm of the animal’s baseline.

We encountered no major bleeding problems or surgical complications while completing the femoral cut down or burr hole with brain death induction. The hemodynamics of brain death (time 0) are outlined in Figs 3 and 4. We noted that although all animals started with slightly different systolic blood pressures, there was a predicable hypertension, followed by hypotension before return to baseline (Fig 3B). A mean SBP of 231.3±29.4mmHg was identified immediately at herniation, then a slow drop in SBP until a nadir at 69.0±23.27mmHg approximately 45 minutes post-brain death (Fig 3A). Finally, we documented a return to baseline by 1-hour post-brain death. The animals exhibited variability in terms of heart rate (HR) response to brain death (Fig 3D). Although most animals became tachycardic and remained tachycardic above their initial baseline, one animal (#9) became bradycardic to 60 bpm and then experienced rebound tachycardia. This animal subsequently developed acute pulmonary edema. When all animals HRs were averaged (Fig 3C), the trend was a slightly delayed tachycardia to 184.4±24.5 bpm 15 minutes after brain death, followed by slow return to a HR approximately 10–15 bpm above the animal’s original baseline by 1 hour post brain death.

#### Maintenance of hemodynamic stability

Utilizing the methods outlined above, we were able to maintain hemodynamic stability throughout brain death with MAP>60 and HR within 20 bpm of the animal’s baseline (Fig 4). In order to accomplish these goals, multiple vasoactive infusions and fluid boluses were administered (Table 4). While three animals required only vasopressin and fluid boluses, three animals required a total of three or more vasoactive agents. The pressor requirements for all animals increased with duration of brain death. The largest vasopressor requirement was noted in the last 1–3 hours of the 20 hour experiment. The average CVP for all animals was 2.7±1.37mmHg and average total volume status at hour 20 was +311.52ml.

**Table 4:** Vasoactive drug use by animal.

Animal	Hours on Vasopressin	Max Dose (Range: 0.0003–0.005un/ kg/min)	Hours on Dopamine	Max Dose (Range: 5-50mcg/kg/min)	Hours on Epinephrine	Max Dose (Range: 0.1–1 mcg/kg/ min)	Hours on Dobutamine	Max Dose (range: 0.5–20 mcg/kg/min)	Fluid Bolus (10ml/kg)
1	10.5	0.003	None	N/A	None	N/A	None	N/A	6
2	11.5	0.005	2	50	0.5	0.1	None	N/A	6
3	13.75	0.002	None	N/A	None	N/A	None	N/A	1
4	14.25	0.004	1	5	None	N/A	None	N/A	3
5	7.5	0.003	None	N/A	None	N/A	None	N/A	3
6	16.25	0.002	None	N/A	None	N/A	None	N/A	5
7	18.5	0.004	8.25	50	1	0.1	None	N/A	4
8	11.5	0.003	7.5	50	3	0.3	3	5	5
9	14	0.003	3.5	30	None	N/A	None	N/A	2

**Vasoactive drug use by animal:** While three animals required only vasopressin and fluid, three animals required a total of three or more vasoactive agents. The pressor requirements for all animals increased with duration of brain death with the largest medication requirement in the last 1–3 hours of the 20 hour experiment

Troponin values were drawn every 6 hours until they peaked. The average peak troponin value was 8.15± 9.83μg/L, with a maximum peak troponin in animal #8 of 27.4 μg/L and minimum peak troponin in animal#5 of only 0.2 μg/L. In addition to troponin elevations, we were able to document ECG changes consistent with myocardial strain/infarction. During the brain death procedure for animal #8, we noted classic ST elevation, followed by T wave inversion characteristic of myocardial ischemia. No significant prolonged arrhythmias were noted in our animals. Five of the nine animals developed sustained tachycardia>20bpm above baseline during the maintenance phase of brain death. To treat this and reduce myocardial oxygen consumption, the animals were administered labetalol at 0.25mg/kg with an average of 1.6±1.9 doses in the 20-hour period. No animals experienced complications or refractory hypotension/bradycardia from this medication.

### Respiratory

**Fig 5:**
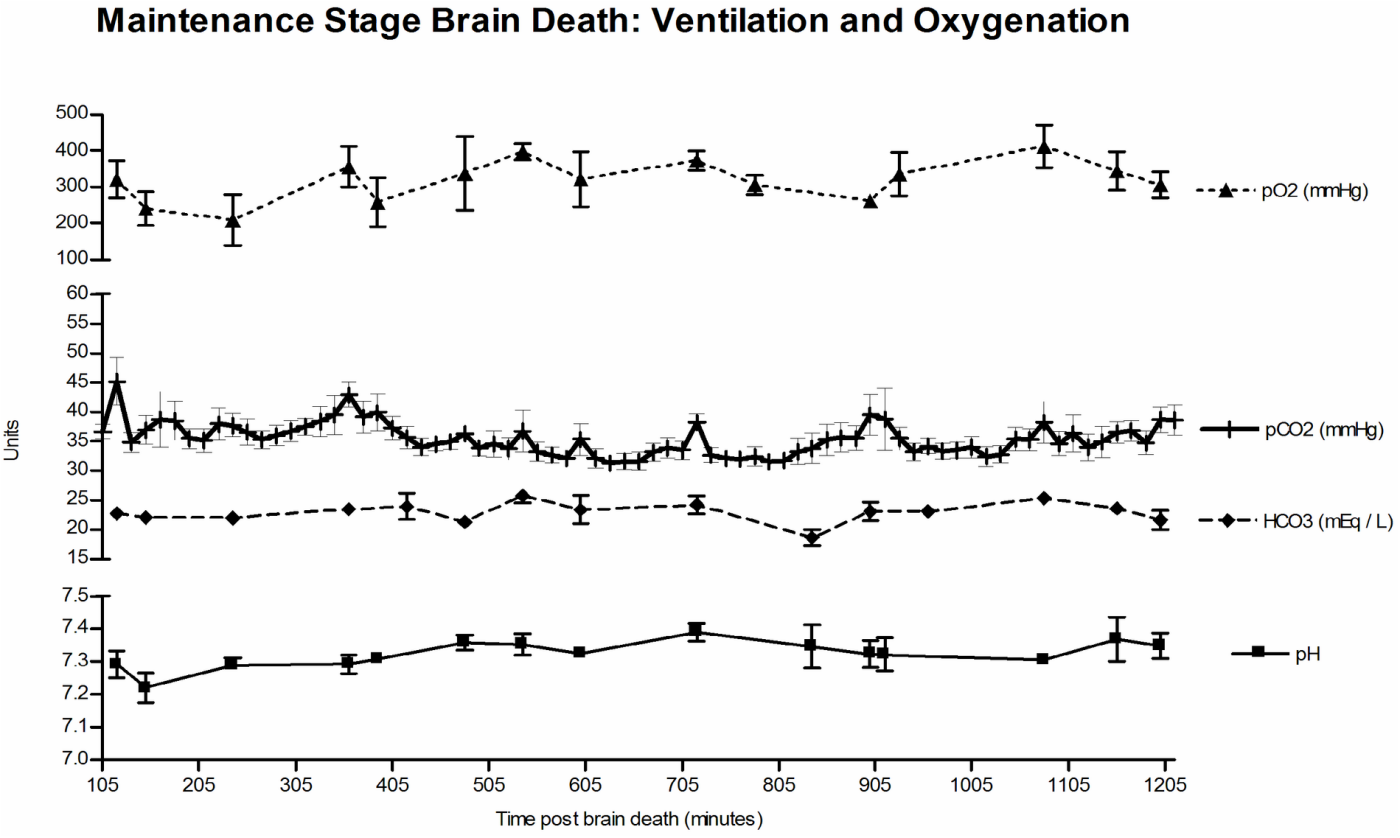
Maintenance of brain death—Ventilation and oxygenation. Using by following serial ABGs and making appropriate ventilator adjustment, we were able to maintain adequate oxygenation and ventilation throughout brain death.

Using the respiratory trouble shooting techniques and ventilator adjustments outlined above, we were able to maintain adequate oxygenation and ventilation throughout the 20-hour maintenance brain death period (Fig 5). We did have four animals with significant secretions requiring inline suctioning, three animals with atelectasis requiring breath holds with the Ambu-bag and one animal that went into pulmonary edema following an episode of bradycardia after induction of brain death. This animal (#9) required inline suction, breath holds, and 2mg/kg IV Lasix, but recovered after approximately 90 minutes with no pO2 level<63mmHg.

### Genitourinary

We documented no evidence of acute renal injury, with average creatinine of 0.59±0.13mg/dL and blood urea nitrogen (BUN) of 14.7±3.84 mg/dL. There was no statistically significant difference between the initial mean creatinine of 0.54±0.22 mg/dL and end mean creatinine of 0.61±0.20 mg/dL (p = 0.141).

### Endocrine

#### Management of DI

The management of DI in our animals is outlined in Table 5. All of the animals experienced some hypernatremia with and average sodium for all animals across all time points of 151.6±5.6 mEq/L and average maximum sodium of 156.6±5.61 mEq/L. All animals except one (#5) required a D5W infusion to treat hypernatremia with an average infusion time of 11.5±5.5 hours.

**Table 5:** Management and severity of diabetes insipidus.

Animal	Hours on Vasopressin	Max Dose (Range: 0.0003–0.005un/kg/min)	Max urine output (ml/kg/hr)	Average urine output (ml/kg/min)	Hours on D5W infusion (h)	Max Sodium mEq/L	Average Sodium mEq/L
1	10.5	0.003	27.5 @ hour 9	9.63±6.8	10	173 @ hour 10.5	163.4 ± 7.40
2	11.5	0.005	14.4 @ hour 5	6.0±3.9	12	164@ hour 11	156.5 ± 1.64
3	13.75	0.002	4.3@ hour 7	2.4±1.03	17.5	151@ hour 5	147.3 ± 2.98
4	14.25	0.004	7.1@ hour 11	2.9 ± 1.77	16	151@ hour 9	148.4 ± 2.75
5	7.5	0.003	3.1@ hour 4	1.4 ± 0.70	0	153 @ hour 15	146.7 ± 2.98
6	16.25	0.002	34.5 @ hour 6	8.8 ±8.29	14	162 @ hour 9	155 ± 3.66
7	18.5	0.004	16.7 @ hour 19	4.9 ± 3.63	14	152 @ hour 9	149.4 ± 3.31
8	11.5	0.003	11.9 @ hour 11	4.4 ± 2.67	14	154 @ hour 9.5	150.7 ± 2.49
9**	14	0.003	8.4@ hour 10	3.4 ± 2.5	6	150 @ hour	147.2 ± 1.79

** Animal received Lasix for pulmonary edema

**Management and severity of diabetes insipidus:** The management of DI in our animals. All of the animals experienced some hypernatremia. All animals except one required a D5W infusion to treat hypernatremia. The peak UO for most animals occurred between hours six and eleven. All but one animal exceeded the 4ml/kg/hr of urine output. Vasopressin infusions were utilized in all animals.

**Fig 6:**
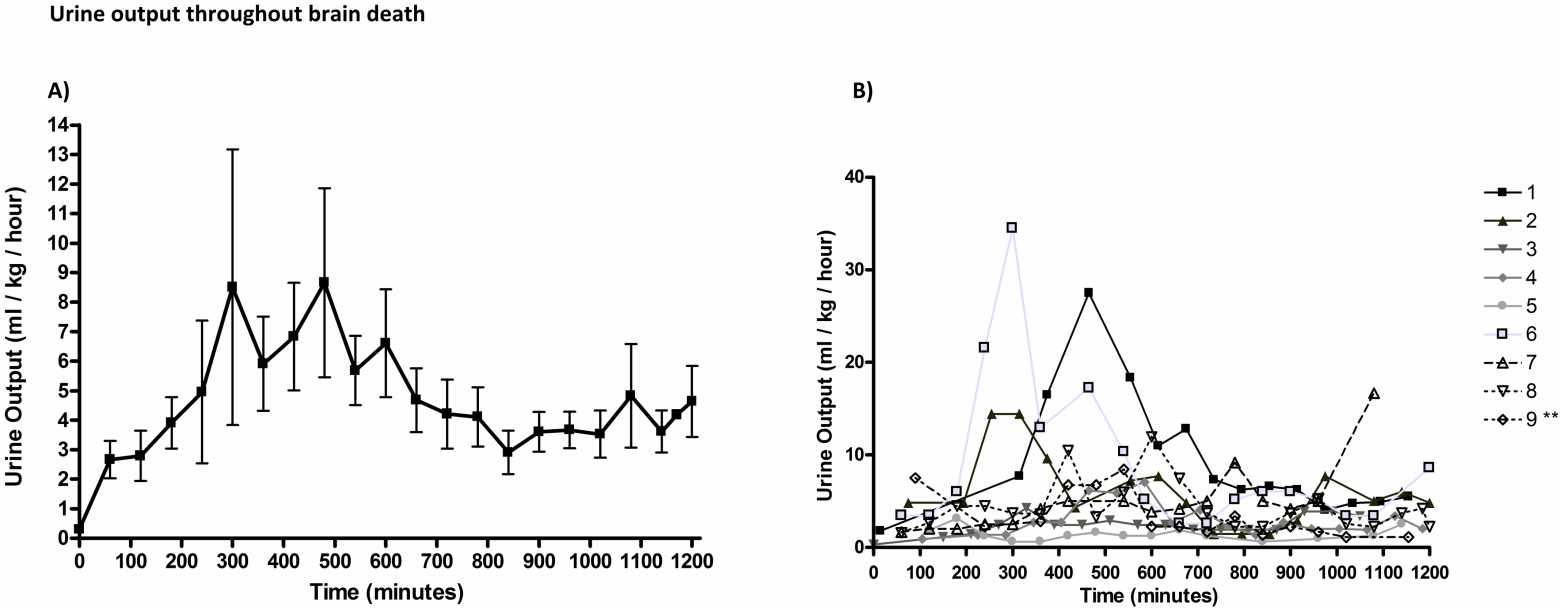
Urine output throughout brain death. Although there was variability among animals (Fig 6B), the average peak UO occurred 5–8 hour after BD induction (Fig 6A). All but one animal exceeded the 4ml/kg/hr of urine output at some time point in the experiment.

The urinary output (UO) values for all animals and trend is summarized in Fig 6. Although there was wide variability among animals (Fig 6B), the average peak UO occurred 5–8 hour after BD induction (Fig 6A). All but one animal exceeded 4ml/kg/hr of urine output at some time point in the experiment. The average hourly urine output for all animals across all time points was 4.87±2.82 ml/kg/hr with an average maximum urine output of 14.2±10.6 ml/kg/hr. Vasopressin infusions were utilized in all animals with an average infusion time of 13.08± 3.25 hours and average maximum dose of 0.003±0.001 units/kg/min.

#### Maintenance of normoglycemia

We did not experience significant difficulty with glucose control even in animals on D5W infusions. The mean glucose for all animals across all time points was 111.2 ± 21.18 mg/dL. We documented only one episode of glucose>200 with average maximum glucose of 157.7±39.4 mg/dL. No animal required insulin to meet our goals of glucose<200 mg/dL.

### Organ procurement

**Fig 7:**
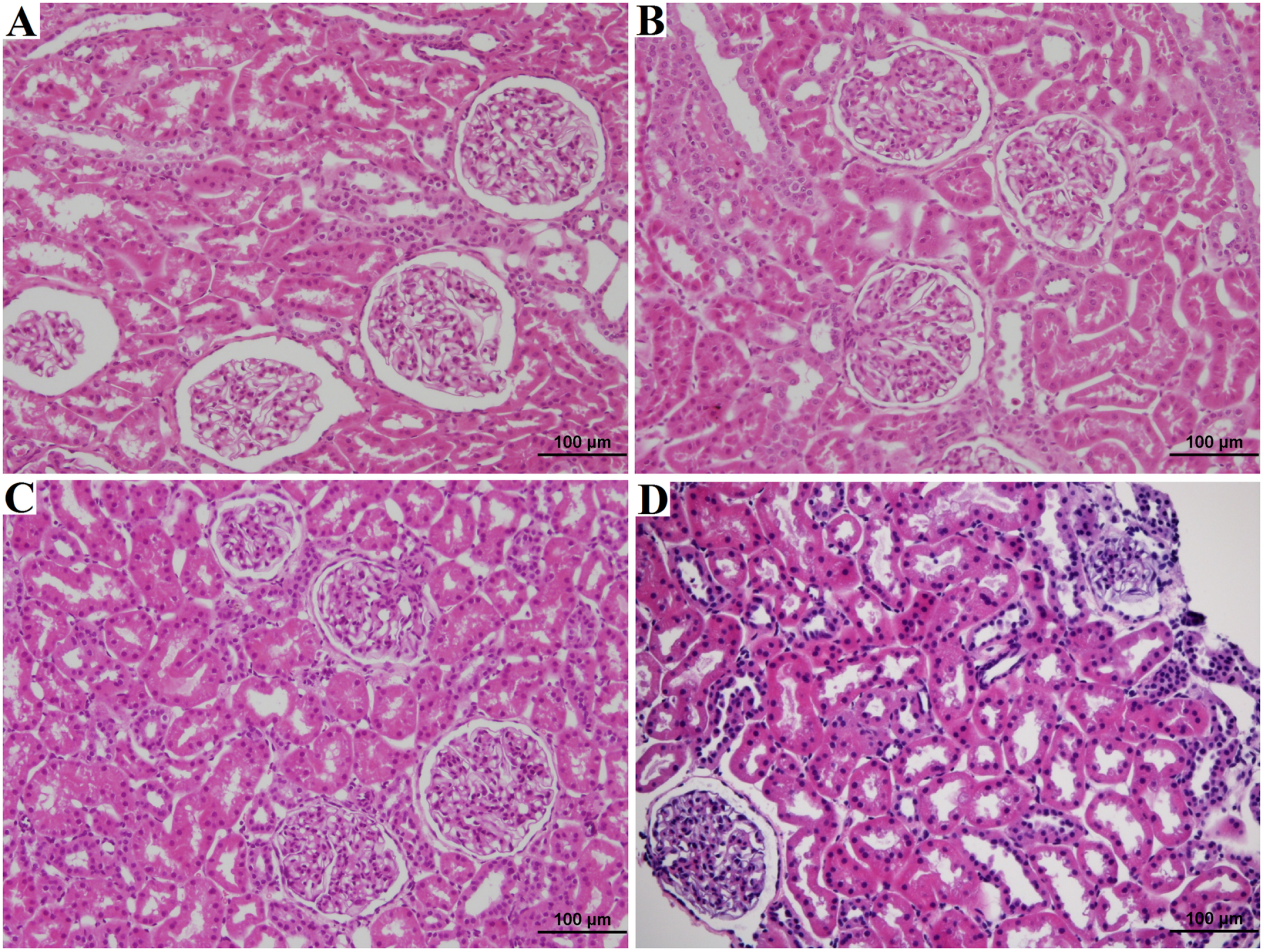
Kidney H&E analysis. Biopsy A shows Kidney H&E of naïve rhesus, age 18 years, diagnosed as normal. Biopsy B shows Kidney H&E of naïve rhesus, age 15 years, diagnosed with mild acute tubular injury, focal mild arteriosclerosis. Biospy C shows Kidney H&E of brain dead rhesus, age 16 years, diagnosed normal. Biopsy D shows Kidney H&E of naïve rhesus, age 19 years, diagnosed with mild acute tubular injury, focal mild arteriosclerosis.

**Fig 8:**
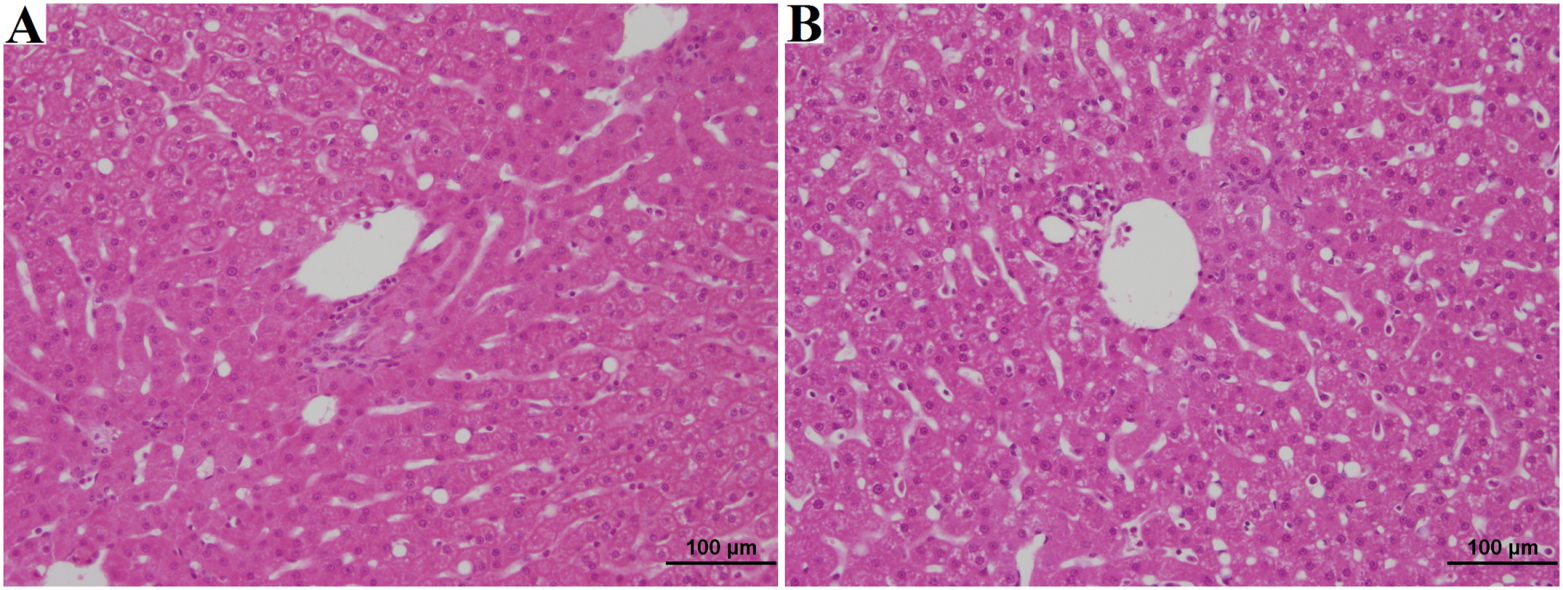
Liver H&E analysis. Biopsy A shows Liver H&E of naïve rhesus, age 15 years, diagnosed 5–10% macrovesicular fatty infiltrates, moderate/marked vacuolization. Biopsy B shows Liver H&E of brain dead rhesus, age 18 years, diagnosed 5–10% macrovesicular fatty infiltrates, moderate vacuolization.

**Fig 9:**
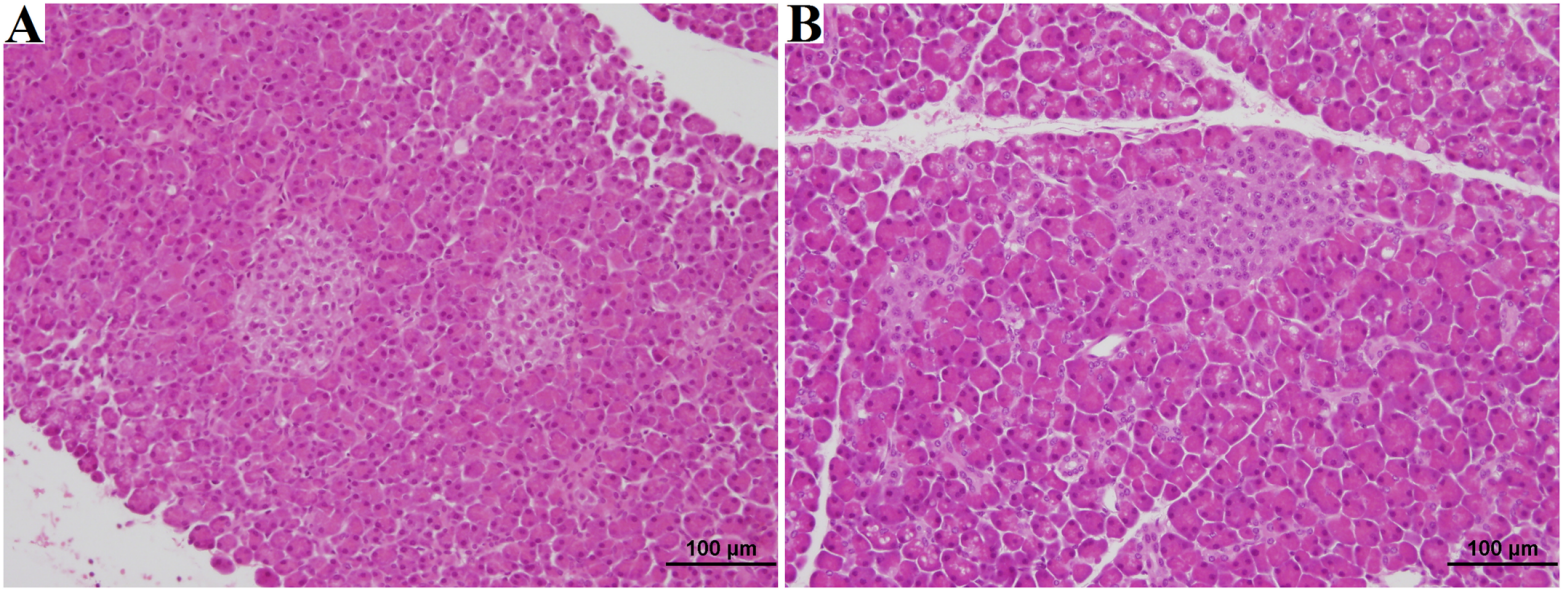
Pancreas H&E analysis. Biopsy A shows H&E of naïve rhesus, age 21 years, diagnosed mild apoptosis, minimal lobular atrophy. Biopsy B shows H&E of brain dead rhesus, age 17 years, diagnosed mild apoptosis, minimal lobular atrophy.

Following organ procurement, core needle biopsies were taken from 17 of the 18 kidneys and wedge biopsies taken from all 9 of the animal’s liver and pancreas. There was no difference (p = 0.23) between the mean age of the brain dead animals (17.9±1.41 years) and naïve control animals (17.7±2.29 years). A full histological summary of the findings are summarized in Table 6. We noted no statistically significant differences (p>0.20) in damage to the renal glomeruli, intersitium, or vasculature. We did note our control naïve animals had more extensive age related renal tubular damage (p<0.01), After applying the Remuzzi score[27], all the naïve animals had organs suitable for single kidney transplant and all brain dead animals had organs suitable for single organ transplant. When evaluating the liver and pancreas wedge biopsies, there were no differences noted in the histological characteristics between naïve and brain dead animals (p>0.06). Representative histological slides from the naïve and brain dead animals are shown in Figs 7–9.

**Table 6:** Histological analysis of brain dead vs. naïve primates.

	Naïve Animals (n = 7)	Brain Death Animals n = 9 for liver/pancreas n = 17 for kidney	P value
Mean Age (years)	17.7±2.29	17.9±1.41	0.23
Kidney Histology			
**Glomeruli** ** Normal Small Focal infarct	7 (100%) 0 (0%)	13 (92.9%) 1 (7.1%)	0.37
**Tubules** Normal Mild acute tubular necrosis Moderate acute tubular necrosis	0 (0%) 2 (28.6%) 5 (71.4%)	6 (35.3%) 11 (64.7%) 0 (0%)	<0.01
**Interstitium** Normal Interstitial Fibrosis	7 (100%) 0 (0%)	16 (94.1%) 1 (5.9%)	0.51
**Vascular** Normal Mild arteriosclerosis	3 (42.9%) 4 (57.1%)	15 (88.2%) 2 (11.8%)	0.20
**Remuzzi Score** 0 to 3 (mild)- OK for single transplant 4 to 6 (moderate)- OK for dual transplant 7 to 12 (severe)- should not be transplanted Unable to score given focal infarct	7 (100%) 0 (0%) 0 (0%) 0 (0%)	17 (100%) 0 (0%) 0 (0%) 1 (5.9%)	0.51
Liver Histology			
**Hepatocyte Borders** Distinct Non-Distinct	5 (71.4%) 2 (28.6%)	9 (100%) 0 (0%)	0.09
**Fatty Infiltrates** < 5% macrovesicular 5–10% macrovesicular 10% macrovesicular	2 (28.6%) 4 (57.1%) 1 (14.3%)	2 (22.2%) 4 (44.4%) 3 (33.3%)	0.68
**Fibrosis** None	7 (100%)	9 (100%)	n/a
**Vacuolization** None Minimal Mild Moderate Moderate/Marked	1 (14.3%) 0 (0%) 0 (0%) 3 (42.9%) 3 (42.9%)	1 (11.1%) 3 (33.3%) 3 (33.3%) 2 (22.2%) 0 (0%)	0.06
**Necrosis** None Scattered Foci Zone 3	5 (71.4%) 0 (0%) 2 (28.6%)	6 (66.7%) 1 (11.1%) 2 (22.2%)	0.65
Pancreas Pathology			
**Apoptosis** None Mild Moderate Marked	1 (14.3%) 5 (71.4%) 1 (14.3%) 0 (0%)	0 (0%) 4 (44.4%) 2 (22.2%) 3 (33.3%)	0.27
**Lobular Atrophy** None Minimal/Mild Moderate	3 (42.9%) 4 (57.1%) 0 (0%)	1 (11.1%) 7 (77.8%) 1 (11.1%)	0.24

** 3 biopsy samples unable to assess glomeruli given no glomeruli in punch biopsy

## Discussion

Significant damage can occur to transplantable organs during the brain death process and, in fact, the conversion rate in United States from potential to actual donors is only 60%[19, 20]. By utilizing our translational model, pharmacological interventions (such as evaluation of new drugs) can be tested in nonhuman primates to understand potential clinical significance and evaluate safety prior to human testing. This manuscript describes in detail the successful development of a rhesus macaque brain death model that mimics clinical practice. Histological analysis of our organs following a 20 hour brain death period demonstrates no significant difference when compared to naïve controls and Remuzzi scores in all brain dead animal’s kidneys demonstrated the quality was sufficient for single organ transplant.

With the exception of one animal, we were able to confirm brain death based on physical exam and apnea challenge in concordance with the recommendations of the American Academy of Neurology. Given that there was one animal too unstable from a respiratory standpoint to undergo brain death confirmation by these means, ancillary testing (cerebral angiogram, EEG, transcranial Doppler) may be necessary for future studies with this model.

We were able to demonstrate reproducible trends of hemodynamic instability immediately after brain death with regards to systolic blood pressure and heart rate. During the initial stage of hypertension and tachycardia, little intervention was needed (lasting<15 minutes in most cases) and this was followed by significant hypotension in most cases. The initial sympathetic surge is caused by release of catecholamines from postganglionic sympathetic nerve endings and can precipitate left ventricular dysfunction, arrhythmias and cardiac stunning[23]. The second stage of brain death is caused from herniation and loss of sympathetic tone causing hypotension[28]. This can be exacerbated if the animal was hypovolemic from being fasted the night before for the surgery. As a result, it is our protocol to pre-hydrate the animals with 20ml/kg subcutaneous 0.9NS twelve hours before brain death. The post-brain death hypotension was initially treated with isotonic intravenous boluses of 10ml/kg to increase preload for the animal. If after two consecutive 10mg/kg boluses (given every 10–15 min), the animal had a mean arterial pressure (MAP<60), a vasoactive agent was started (dopamine or vasopressin) and up to 2 additional bolus were given if needed (total of 40ml/kg). It is well documented that adequate volume expansion and resuscitation is associated with decreased IL-6 and TNF, leading to increased transplant utilization and graft survival[25].

During the maintenance phase of brain death, we were more restrictive with our use of IV fluid boluses in order to prevent complications of fluid overload and hemodilution of research samples. Our goals were a net volume status of less than +250ml and CVP<6 as studies show restrictive fluid management helps avoid volume overload and lung neurogenic edema without compromising kidney graft survival or rates of delayed graft function (DGF) [26][29]. Fluid resuscitation with colloids (albumin and hydroxyethyl starch) should not be used as they can cause acute kidney injury, coagulopathies and compromised graft function[26]. Packed red blood cells are not indicated in donor management unless hemoglobin drops below 7 g/dL, which was not the case in any of our experiments[26].

During the maintenance phase of brain death, we were able to maintain MAP>60mmHg and HR within 20bpm of the animal’s baseline with use of vasoactive drugs and volume replacement. Approximately 80% of human organ donors require some vasopressor support[20], so it is not surprising that we documented need for these medications in our animal model.

In our model, vasopressin infusion was our first line choice in most cases, given its ability to augment blood pressure by improving the vasodilatory shock associated with brain death and treat DI. The benefit of vasopressin is that it is both a potent vasoconstrictor of vascular smooth muscle via V1 receptors, and it also works in the renal collecting system to counteract the profound diuresis found in DI secondary to the lack of endogenous anti-diuretic hormone. Studies have found the use of vasopressin in donor treatment can decrease use of other vasoactive agents and increase rates of successful organ recovery in patients with DI[29]. In fact, the clinical use of vasopressin decreases the need for more harmful catecholamines, such as epinephrine or norepinephrine, and improves cardiac performance[26]. Dopamine should be a second line drug given its ability to be immunomodulatory and attenuate the inflammatory response[19]. Furthermore, it can protect against ischemia/reperfusion injury by induction of heme oxygenase-1 enzyme[26, 30].

Third line and forth line drugs in our model included dobutamine and epinephrine and were only needed in three of the nine animals. Epinephrine was chosen over norepinephrine, but either could be used. Admittedly, norepinephrine is more studied in the human literature. It is important to note that either of these alpha agonists can lead to increased pulmonary capillary permeability and coronary and mesenteric vasoconstriction and should be used in the lowest dose possible for the shortest duration possible[25, 26]. Dobutamine is traditionally only shown to have value in patients with impaired cardiac function. In humans, this is measure by ejection fraction less than 45% on echocardiography[26]. Given that echocardiograms are unavailable to us, we used surrogates in terms of troponin and ECG changes indicative of ischemia. We documented a wide range of troponin changes in our animals with peaks from 0.22–27.4 μg/L. Animals with higher troponin levels were, on average, more hemodynamically unstable. It is important to note that fluid overload in these animals can precipitate heart failure and pulmonary edema. In addition, the judicious use of nonselective beta blockade in animals with evidence of significant cardiac injury can reduce the myocardial oxygen consumption and be cardioprotective. All the animals in our model were over the age of 16 years, so the impact of brain death on the myocardial function of younger animals has not been assessed in this experiment.

We did not have significant problems with oxygenation and ventilation in our animal model. The most frequent complications encountered were secretions, mucus plugs, and atelectasis which could be managed by breath holds with Ambu-bag and PEEP valve or inline suction/lavage. We did encounter one animal (animal #9) who had severe respiratory compromise directly after induction of brain death. The etiology for this was most likely due to either neurogenic pulmonary edema or increased interstitial pressure from profound hypertension without expected tachycardia. Neurogenic pulmonary edema is a phenomenon which occurs in about 20% of severe traumatic brain injuries within minutes to hours[31]. Although the mechanism of neurogenic pulmonary edema is not fully understood, it is thought to be a combination of changes in capillary hydrostatic pressure and pulmonary capillary permeability[32]. Another possible etiology for this animal’s acute pulmonary deterioration was increased pulmonary interstitial pressure and insufficient cardiac output. Unlike all the other animals in our model who exhibited tachycardia and hypertension with brain death, this animal became bradycardic to 60bpm and hypertensive. The etiology of this bradycardia could have been a vagal response from the endotracheal tube or a Cushing response. The increased blood pressure without reflex tachycardia would have increased the pressure within the pulmonary vasculature, leading to leakage of fluid into the interstitum and alveoli.

It is important to not underestimate the profound effect brain death has on the endocrine system. The entire hypothalamic-pituitary system is impacted leading to derangements in thermoregulation, thyroid, adrenal, posterior pituitary hormones, and glucose control. Using a forced air warming blanket (BearHugger) and esophageal temp probe, we were able to maintain normothermia. Mild hypothermia is preferred over hyperthermia given donor hypothermia has been associated with improved transplant outcomes[33]. That being said, profound hypothermia, can exacerbate myocardial depression and arrhythmias[25].

We documented all but one animal (89%) exhibited evidence of DI (urine output >4ml/kg/hr with hypernatremia). This is consistent with the incidence of this disease in 80% of human donors [20]. DI occurs as a result of damage to the posterior pituitary, which results in decreased or even undetectable levels of arginine vasopressin (also known AVP, or antidiuretic hormone)[26]. This hormone stimulates the V1 receptor in vascular smooth muscle causing vasoconstriction, as well as, stimulating V2 receptors which cause increased fluid retention in the renal collecting system[29]. A decrease in plasma AVP levels causes the hallmarks of DI: polyuria (urine outputs>4ml/kg/hour), hypernatremia, dehydration, and hypotension. Continuous vasopressin infusions provide a synthetic replacement for the lost endogenous hormone. These infusions also have the benefit of providing vasoconstriction to improve systemic vascular resistance. Desmopressin was not used given it is only indicated in circumstances of hypernatremia without hypotension[25, 26]. In addition, it is important to replace lost volume with isotonic fluid boluses as guided by your CVP and hourly net fluid volume calculations to prevent dehydration and contraction alkalosis. Even with appropriate use of vasopressin infusions and volume resuscitation, animals may develop hypernatremia from their DI. We found in our first animal that if vasopressin and D5W infusion are delayed until after the urine output is consistently >10ml/kg/hr and sodium is >155, it is difficult to control the hypernatremia. As a result, this animal had our highest sodium of 173 mEq/L and highest UO of 27.5ml/kg/hr. We subsequently started our D5W infusions with Na>150mEq/L. Human literature has shown that donor sodium levels of greater than 155 mEq/L in liver transplant patients and greater than 170 in heart transplant patients are associated with worse outcomes[25].

The evidence on thyroid replacement in donor treatment is controversial with two recent meta-analyses showing no cardiovascular benefits to thyroid supplementation[25]. The changes in thyroid function in donors are more indicative of a euthyroid sick syndrome than a hypothyroid state [26, 34]. It is for these reasons that we did not supplement thyroid hormone in our model. Adrenal insufficiency in donors has also been documented in 76–87% of cases and can potentially lead to hemodynamic compromise[25]. Unfortunately the literature has not completely supported the benefit of high dose steroids with regards to hemodynamic benefits or anti-inflammatory effects[22, 25]. That being said, there are some observational studies to support this theory and administration of 15mg/kg methylprednisolone is still recommended by many critical care societies and can be added to a donor animal model if so desired[25, 26]. Finally, hyperglycemia is a problem seen in a significant number of human donors, but was not seen in our animal model.

## Conclusion

The development of a primate brain death model has significant potential to advance not only transplant research, but also the understanding of the pathophysiologic changes that occur in brain death and severe traumatic brain injury. Our model offers the opportunity for researchers to have translational animal testing prior to human clinical trials of both donor and recipient transplantation drugs and therapies.
